# Comparison of Intestinal Microbes in Noninfectious Anterior Scleritis Patients With and Without Rheumatoid Arthritis

**DOI:** 10.3389/fmicb.2022.925929

**Published:** 2022-06-09

**Authors:** Mengyao Li, Li Yang, Liangliang Zhao, Feng Bai, Xiaoli Liu

**Affiliations:** Ophthalmologic Center of the Second Hospital, Jilin University, Changchun, China

**Keywords:** intestinal microbes, scleritis, rheumatoid arthritis, noninfectious anterior scleritis, *Turicibacter*, *Romboutsia*, *Atopobium*, *Coprobacillus*

## Abstract

We compared intestinal microbes in anterior noninfectious scleritis patients with and without rheumatoid arthritis. Active noninfectious anterior scleritis patients without other immune diseases (G group, 16 patients) or with active rheumatoid arthritis (GY group, seven patients) were included in this study. Eight age- and sex-matched healthy subjects served as controls (N group). DNA was extracted from fecal samples. The V3-V4 16S rDNA region was amplified and sequenced by high-throughput 16S rDNA analysis, and microbial contents were determined. A significant decrease in species richness in the GY group was revealed by α- and β-diversity analyses (*p* = 0.02 and *p* = 0.004, respectively). At the genus level, 14 enriched and 10 decreased microbes in the G group and 13 enriched and 18 decreased microbes in the GY group were identified. Among them, four microbes were enriched in both the G and GY groups, including *Turicibacter*, *Romboutsia*, *Atopobium*, and *Coprobacillus*. Although two microbes (*Lachnospiraceae_ND3007_group* and *Eggerthella*) exhibited similar tendencies in the G and GY groups, changes in these microbes were more significant in the GY group (*p* < 0.05). Interaction analysis showed that *Intestinibacter*, *Romboutsia*, and *Turicibacter*, which were enriched in both the G and GY groups, correlated positively with each other. In addition, nine microbes were decreased in the GY group, which demonstrates a potential protective role for these microbes in the pathogenesis of scleritis *via* interactions with each other.

## Introduction

Scleritis is an immune-mediated disease with initial symptoms of red eyes and pain. Scleritis can be divided into anterior scleritis and posterior scleritis according to anatomical location. Furthermore, anterior scleritis can be divided based on clinical manifestations into nodular anterior scleritis, diffuse anterior scleritis and necrotizing anterior scleritis. Overall, the pathogenesis of scleritis has not been completely elucidated. Although the immune response and infection are considered the main two causes of anterior scleritis, scleritis caused by direct infection of the sclera by pathogenic microorganisms such as bacteria is relatively rare. More than 50% of scleritis cases are associated with immune-mediated diseases, including rheumatoid arthritis and antineutrophil cytoplasmic antibody (ANCA)-associated granulomatosis (GPA; Vergouwen et al., 2020). Among them, rheumatoid arthritis is the most common immune-mediated disease associated with scleritis, and 8%–15% of patients with scleritis have rheumatoid arthritis (RA; Promelle et al., 2021). It has been reported that the level of anti-cyclic citrullinated peptide antibody in RA patients correlates strongly with the severity of ocular manifestations, including scleritis (Vignesh and Srinivasan, 2015). The mechanism of rheumatoid arthritis-associated scleritis is unclear and may be related to the similar structure between the synovium and sclera. Indeed, cells infiltrating the synovium of patients with RA and the sclera of patients with scleritis are similar (Wakefield et al., 2013). Furthermore, HLA-DR4, HLA-DR1, HLA-DR13, and HLA-DR15 are significantly associated with scleritis complicated with RA (Karami et al., 2019).

Intestinal microbes are closely related to the occurrence of RA. However, the role of intestinal microbiota in the pathogenesis of scleritis has not been reported, and whether the intestinal microbiota involved in RA is also related to scleritis is still unknown. Therefore, we investigated the relationship between intestinal microbes and scleritis by comparing intestinal microbes between noninfectious scleritis patients with and without RA. Our results showed four bacterial genera to be enriched in noninfectious anterior scleritis patients both with and without RA, including *Coprobacillus*, *Romboutsia*, *Atopobium*, and *Turicibacter*. In addition, the abundances of three microbes (*Candidatus_Stoquefichus*, *Anaeroplasma*, and *Lactococcus*) were altered in noninfectious anterior scleritis patients without other immune-mediated diseases, and the abundances of nine microbes (*Eubacterium_eligens_group*, *Odoribacter*, *Family_XIII_UCG-001*, *Ruminiclostridium_9*, *Ruminococcaceae_UCG-003*, *Ruminococcaceae_UCG-009*, *Eubcterium_rectale_group*, *Roseburia*, and *Catabacter*) were changed in noninfectious anterior scleritis patients with RA. All these results suggest that intestinal microbes have coexisting identical and distinct roles in the development of scleritis in patients with and without RA.

## Materials and Methods

### Participants

Thirty-one individuals were enrolled in the study, including 16 patients with active noninfectious anterior scleritis (G group, average age: 56.1 ± 7.8 years, male/female: 0.14:1), seven patients with active noninfectious anterior scleritis combined with active RA (GY group, average age: 59.7 ± 10.1 years, male/female: 0.16:1), and eight healthy controls without immune-mediated diseases (N group, average age: 56.1 ± 10.5 years, male/female: 0.14:1). Healthy controls consisted of family members from scleritis patients and age-related cataract patients without immune-mediated diseases. There was no significant difference in age or sex among the three groups in this study. The inclusion criteria for individuals enrolled in the study were as follows: (1) without other immune system diseases, such as ulcerative colitis, systemic lupus erythematosus, or Crohn’s disease; (2) without infectious disease; and (3) patients in the active stage of disease and not taking any medications. The diagnosis of noninfectious anterior scleritis was based on characteristic clinical manifestations, including ocular tenderness to touch, painful inflammation radiating to the forehead, edema affecting episcleral and scleral tissues, and injections of both the superficial and deep episcleral vessels (Dutta Majumder et al., 2020). The diagnosis of active RA was based on the American College of Rheumatology/European League Against Rheumatism 2010 criteria for RA, including confirmed presence of synovitis in at least one joint, absence of an alternative diagnosis better explaining the synovitis, and a total score of 6 or greater (of a possible 10) from the individual scores in the following four domains: number and site of involved joints (range 0–5), serological abnormality (range 0–3), elevated acute-phase response (range 0–1), and symptom duration (two levels; range 0–1; Aletaha et al., 2010). Informed consent was obtained from all subjects. This study met the requirements of the Declaration of Helsinki and was approved by the Clinical Ethics Committee.

### Fecal Sample Collection and DNA Extraction

Feces were collected from patients with anterior scleritis and healthy controls admitted to the Ophthalmologic Center of the Second Hospital between July 2018 and December 2019 and stored at ~80°C for analysis. DNA extraction from feces was performed according to E.Z.N.A. Stool DNA Kit (Omega Bio-Tek, Norcross, GA, United States) following the manufacturer’s instructions. The quality of the extracted DNA was assessed by 1% agarose gel electrophoresis and spectrophotometry (260/280 nm optical density ratio). The target sequence that needed to be amplified was introduced in the sequencing of the 16S ribosomal RNA (rRNA) gene amplicon.

### Sequencing of 16S rRNA Gene Amplicons

The extracted DNA was sent to Beijing Aoweisen Gene Technology Co., Ltd. (Beijing, China). We referred to previously reported experimental methods to amplify the target gene (Li et al., 2022), as follows. We detected the DNA using the Illumina MiSeq PE300 platform (Santiago, CA, United States) and used the universal primers 338F (5-ACTCCTACGGGAGGCAGCAG-3) and 806R (5-GGACTACNNGGG TATCTAAT-3) to amplify the V3 to V4 16S ribosomal DNA (rDNA) region. Next, we amplified the target sequence by polymerase chain reaction.

### Sequence Analysis

We used previously reported experimental methods to analyze the sequence (Li et al., 2022) as follows. Paired-end sequencing of the target sequence was performed using the Illumina MiSeq platform, and QIIME (Professor Gregory Caporaso, Flagstaff, United States; version 1.8.0) was used to filter, split, and remove chimeras. Sequences with scores less than 20 or that had base ambiguity, primer mismatch, or a length less than 150 bp were excluded. The sequences were clustered and grouped as operational taxonomic units (OTUs) based on barcodes. We set the OTU similarity to 97%, and we matched every OTU to corresponding species classification information by comparison with the Silva database. Then, we calculated the microbial α-diversity, including the Shannon, abundance-based coverage estimator (ACE), and Chao1 indices, in Mothur (version 1.31.2, Professor Patrick Schloss; MI, United States). The species communities of each sample were compared, and β-diversity was calculated by UniFrac. Clustering was performed using pheatmap in TBtools (version 1.098652, Chengjie, Chen; Guangzhou, China) based on the weighted UniFrac distance. The data are presented based on the row scale. Raw reads were uploaded to the Sequence Read Archive (SRA) database on the National Center for Biotechnology Information website. The BioProject ID is PRJNA836534.

### Statistical Analysis

To explore differences between groups, Mothur software (version 1.31.2) was used to perform Metastats analysis, and a *p* < 0.05 indicated significance. Linear discriminant analysis (LDA) coupled with effect size (LEfSe) was performed using Glaxy (The Huck Institutes of the Life Sciences, The Institute for CyberScience at Penn State, and Johns Hopkins University, United States). Only data with a *p* < 0.05 and a log LDA score > 2 are reported. Spearman correlation analysis was used as the mapping parameter of the correlation diagram. We visualized and analyzed the network using the personalbio platform (Zikui Sun, Shanghai, China), and the correlation was | R | > 0.6, *p* < 0.05.

## Results

### Microbiome Species Diversity and Number of Samples Sequenced

**Figure 1:**
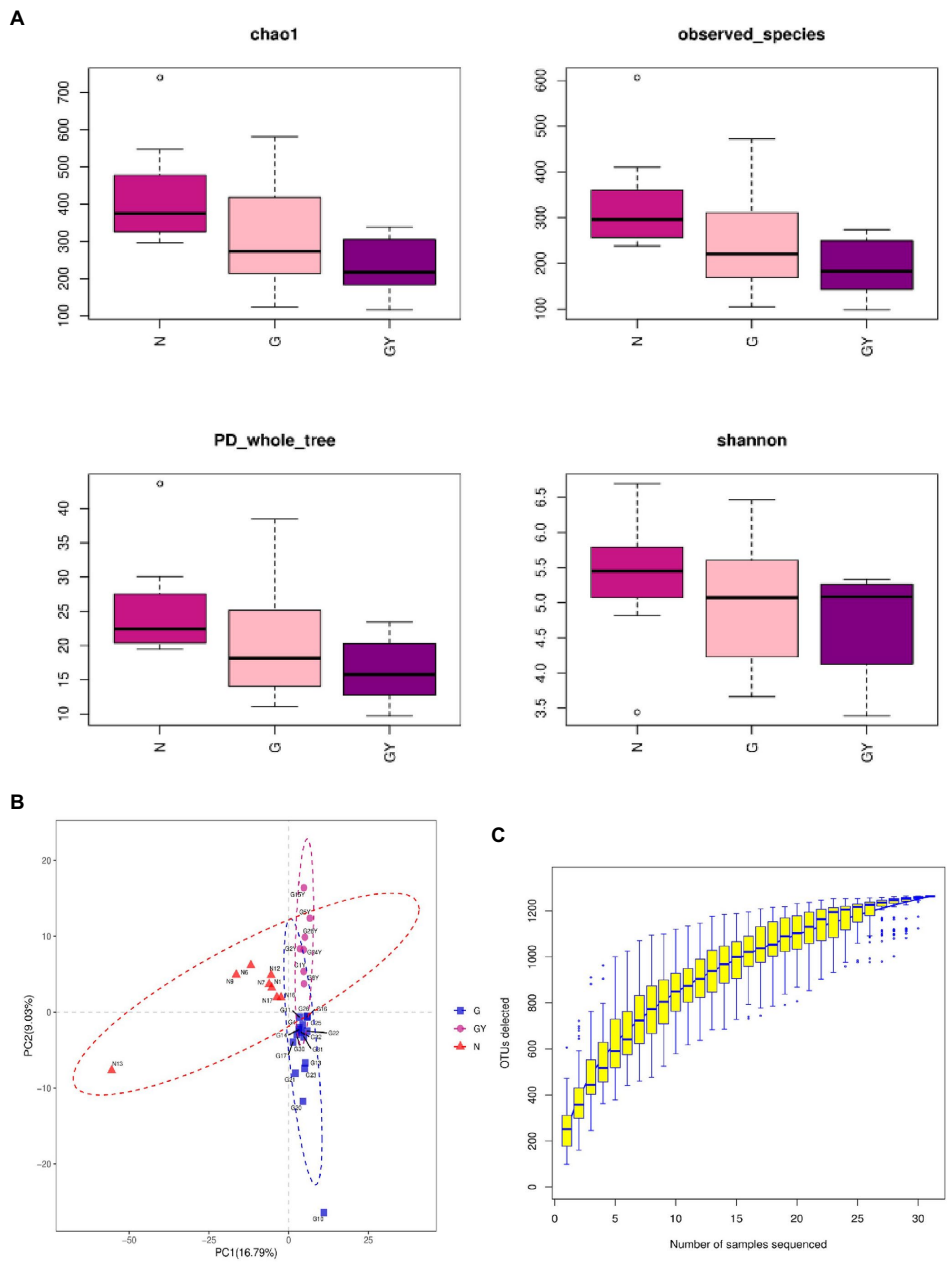
**(A)** α-Diversity using the Chao1, observed OTUs, PD_whole_tree, and Shannon measures for patients with active noninfectious anterior scleritis, patients with active noninfectious anterior scleritis combined with active rheumatoid arthritis, and healthy controls. **(B)** β-Diversity was assessed by analysis of molecular variance (ANOVA) in patients with active noninfectious anterior scleritis, patients with active noninfectious anterior scleritis combined with active rheumatoid arthritis, and healthy controls. **(C)** Rarefaction curves of the gut microbes from 16 patients with active noninfectious anterior scleritis, seven patients with active noninfectious anterior scleritis combined with active rheumatoid arthritis, and 11 healthy controls.

The α-diversity (Chao1, observed_species, PD_whole_tree, and Shannon) and β-diversity [analysis of molecular variance (AMOVA)] of the intestinal microbes in each group were analyzed (Figures 1A,B). According to α-diversity analysis, the species in the N group were most abundant, followed by the G and GY groups, and there were significant differences in species richness between the N and GY (*p* = 0.02) groups. Differences in species richness between the N and GY (*p* = 0.004) groups were detected by β-diversity analysis, and differences between the G and GY (*p* = 0.04) groups were significant.

We examined the number of reads sampled and found that the number of OTUs did not further increase with an increase in the number of samples sequenced. Thus, the sequencing depth and coverage were sufficient to cover the total diversity of the microbiomes examined (Figure 1C).

### Changes in Intestinal Microbes in Noninfectious Anterior Scleritis Patients Without Other Immune-Related Diseases

**Figure 2:**
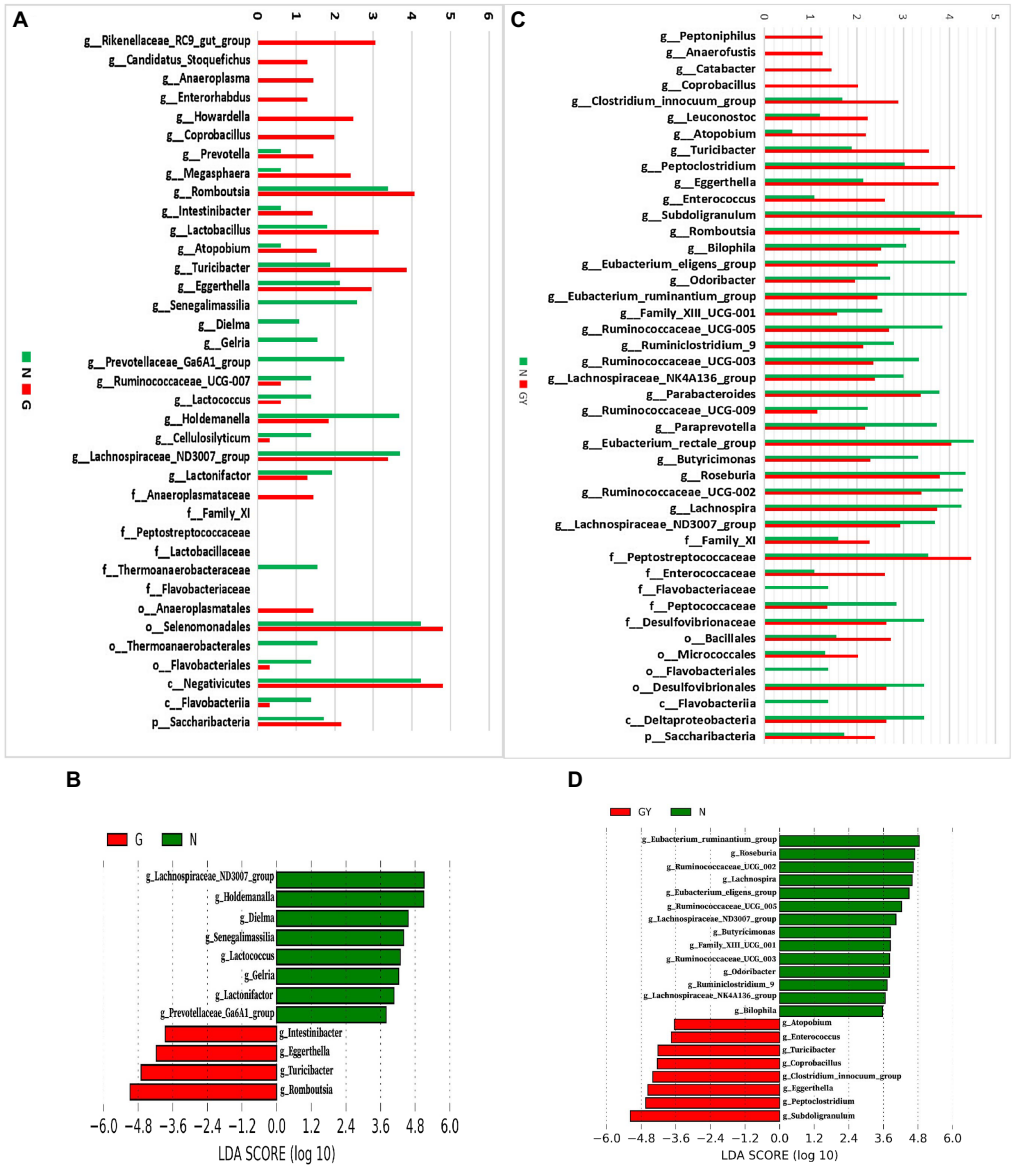
**(A)** Relative abundance of microbes in 16 patients with active noninfectious anterior scleritis (G) and 11 healthy controls (N); **(B)** Linear discrimination analysis (LDA) effect size (LEfSe) analysis results comparing active noninfectious anterior scleritis patients (G) and 11 healthy controls (N); **(C)** Relative abundance of microbes in seven patients with active noninfectious anterior scleritis combined with active rheumatoid arthritis (GY) and 11 healthy controls (N); **(D)** LDA LEfSe analysis results comparing patients with active noninfectious anterior scleritis combined with active rheumatoid arthritis (GY) and 11 healthy controls (N). The content of microbes **(A,C)** was increased by 10^6^-fold; the logarithm was taken, and the base number was 10. LDA scores **(B,D)** (log10) > 2 are listed.

To investigate microbes involved in the pathogenesis of noninfectious anterior scleritis, we screened microorganisms with significantly different abundances between healthy people (Group N) and patients with noninfectious anterior scleritis without RA (Group G; Figure 2A).

At the genus level, 14 enriched and 10 decreased microbes were detected in the G group. Among these enriched microbes, six were only detected in Group G, including *Rikenellaceae_RC9_gut_group*, *Candidatus_Stoquefichus*, *Anaeroplasma*, *Enterorhabdus*, *Howardella*, and *Coprobacillus*. Eight microbes were detected in both Group G and Group N, but the contents in the former were higher, including *Prevotella*, *Megasphaera*, *Romboutsia*, *Intestinibacter*, *Lactobacillus*, *Atopobium*, *Turicibacter*, and *Eggerthella*. Among the 10 microbes with decreased abundance, 4 were only detected in Group N: *Senegalimassilia*, *Dielma*, *Gelria*, and *Prevotellaceae_Ga6A1_group*. Six microbes were detected in both Group G and Group N, but the content in Group G was lower, including *Ruminococcaceae_UCG-007*, *Lactococcus*, *Holdemanella*, *Cellulosilyticum*, *Lachnospiraceae_ND3007_group*, and *Lactonifactor*.

To identify possible biomarkers, LEfSe analysis was performed to examine different microbial features between active noninfectious anterior scleritis patients and healthy controls. The results were similar to those above, with enrichment of *Romboutsia*, *Turicibacter*, and *Eggerthella* and a decrease in *Lachnospiraceae_ND3007_group*, *Holdemanella*, and *Dielma* in the G group (Figure 2B).

At the family level, *Anaeroplasmataceae*, *Family_XI*, *Peptostreptococcaceae*, and *Lactobacillaceae* were increased and *Thermoanaerobacteraceae* and *Flavobacteriaceae* decreased. At the order level, *Anaeroplasmatales* and *Selenomonadales* were enriched, whereas *Thermoanaerobacterales* and *Flavobacteriales* were reduced. At the class level, *Negativicutes* was increased and *Flavobacteriia* decreased. At the phylum level, *Saccharibacteria* showed enrichment.

### Changes in Intestinal Microbes in Noninfectious Anterior Scleritis Patients With RA

Next, we screened microbes with significantly different abundances in noninfectious anterior scleritis patients with RA (GY group; Figure 2C).

At the genus level, 13 enriched and 18 decreased microbes were detected in the GY group. Among the enriched microbes, four were only detected in the GY group, including *Peptoniphilus*, *Anaerofustis*, *Catabacter*, and *Coprobacillus*. Nine microbes were detected in Group GY and Group N, but the content in Group GY was higher than that in Group N, including *Clostridium_innocuum_group*, *Leuconostoc*, *Atopobium*, *Turicibacter*, *Peptoclostridium*, *Eggerthella*, *Enterococcus*, *Subdoligranulum*, and *Romboutsia*. The 18 microbes with decreased abundance in the GY group included *Bilophila*, *Eubacterium_eligens_group*, *Odoribacter*, *Eubacterium_ ruminantium_group*, *Family_XIII_UCG-001*, *Ruminococcaceae_UCG-005*, *Ruminiclosridium_9*, *Ruminococcaceae_UCG-003*, *Lachnospiraceae_ NK4A136_group*, *Parabacteroides*, *Ruminococcaceae_UCG-009*, *Paraprevotella*, *Eubacterium_rectale_group*, *Butyricimonas*, *Roseburia*, *Ruminococcaceae_UCG-002*, *Lachnospira*, and *Lachnospiraceae_ND3007_group*. The *Intestinibacter* content in the GY group was higher than that in the N group, but the difference was not significant.

In LEfSe analysis of was performed to examine microbial features between noninfectious anterior scleritis patients with RA and healthy controls. Similar findings were found, including *Subdoligranulum*, *Peptoclostridium*, and *Eggerthella* enrichment and decreases in *Eubacterium_ruminantium_group*, *Roseburia*, and *Ruminococcaceae_ UCG-002* in the GY group (Figure 2D).

At the family level, *Family_XI*, *Peptostreptococcaceae*, and *Enterococcaceae* were enriched in the GY group, but *Flavobacteriaceae*, *Peptococcaceae*, and *Desulfovibrionaceae* were decreased. *Bacillales* and *Micrococcales* were enriched at the order level, whereas *Flavobacteriales* and *Desulfovibrionales* were decreased. *Flavobacteriia* and *Deltaproteobacteria* were decreased at the class level, and *Saccharibacteria* was enriched at the phylum level (Figure 2C).

### Comparison of Intestinal Microbes Between the Two Groups of Patients

**Figure 3:**
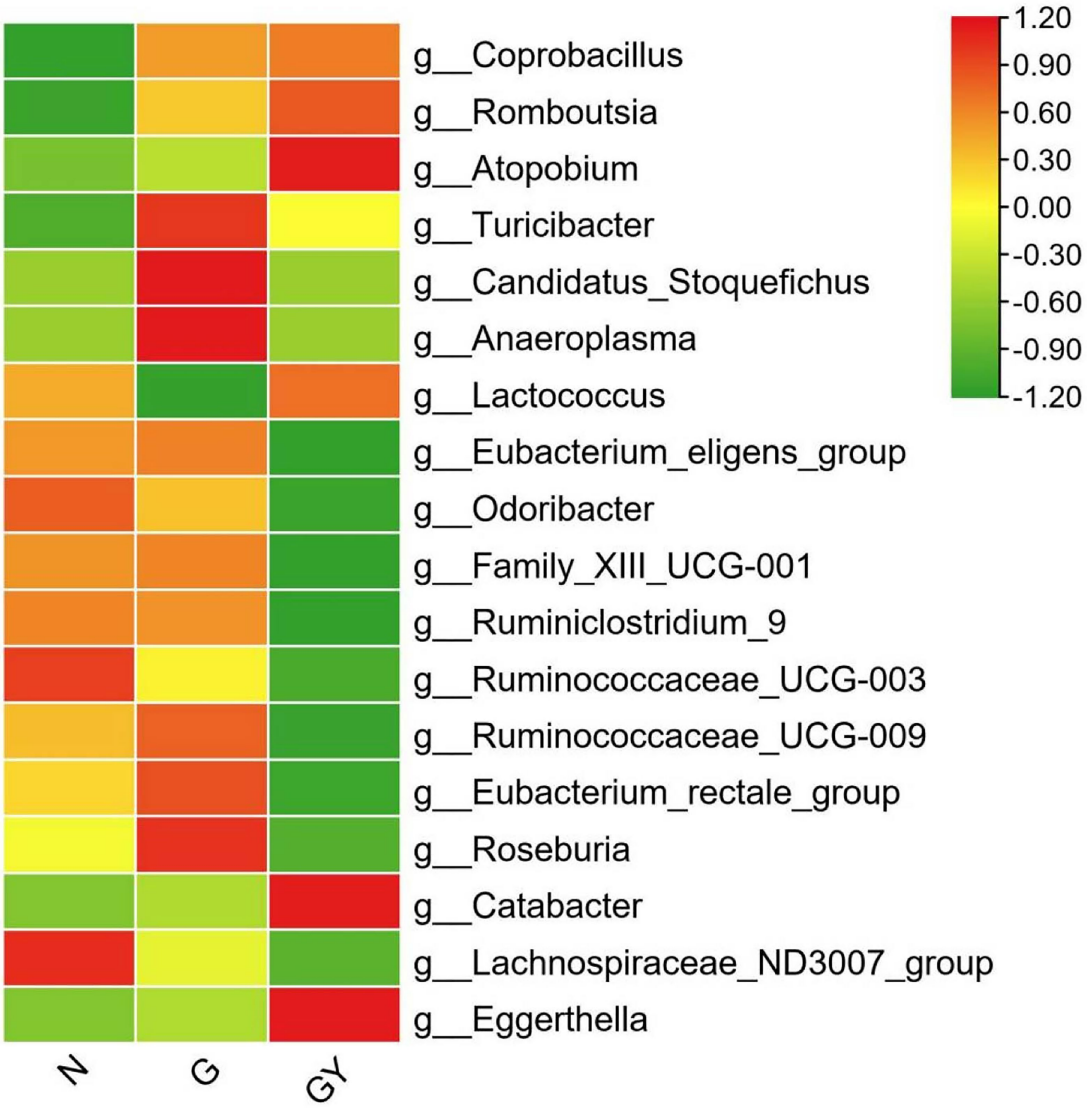
Relative abundance of microbes from 16 patients with active noninfectious anterior scleritis (G), seven patients with active noninfectious anterior scleritis combined with active rheumatoid arthritis (GY), and 11 healthy controls (N). The content of microbes is presented based on the row scale.

To identify microbes related to noninfectious scleritis, we compared the three groups and divided microorganisms into four types (Figure 3).

#### Type A

Four microbes were enriched in both Groups G and GY, including *Coprobacillus* (both *p* < 0.05), *Romboutsia* (both *p* < 0.01), *Atopobium* (both *p* < 0.05), and *Turicibacter* (both *p* < 0.01). The abundances of these microbes were significantly different between Groups N and G or GY, but there was no significant difference between Groups G and GY. We consider these microbes to be related to the pathogenesis of scleritis.

#### Type B

Microbes were enriched or decreased only in the G group, and not in the GY group, including *Candidatus_Stoquefichus*, *Anaeroplasma*, and *Lactococcus*. We consider that these microbes may be specifically related to scleritis pathogenesis.

#### Type C

Microbes were enriched or decreased only in the GY group, and not in Group G, including *Eubacterium_eligens_group*, *Odoribacter*, *Family_XIII_UCG-001*, *Ruminiclostridium_9*, *Ruminococcaceae_UCG-003*, *Ruminococcaceae_UCG-009*, *Eubacterium_rectale_group*, *Roseburia*, and *Catabacter*. We consider these microbes to be related to the pathogenesis of RA or the shared pathogenesis pathway of scleritis and RA.

#### Type D

Two microbes (*Lachnospiraceae_ND3007_group*, *Eggerthella*) that exhibited similar abundances in both the G and GY groups. The abundance of *Lachnospiraceae_ND3007_group* was decreased and that of *Eggerthella* enriched. The changes in both the G and GY groups were significant compared with those in the N group, but the *Eggerthella* content was higher and the *Lachnospiraceae_ND3007_group* content lower in the GY group than in the G group. Interestingly, these two microbes also exhibited similar changes between patients with active VKH and patients with scleritis (Li et al., 2022). This result suggests that these two microbes may be nonspecifically involved in various immune-related eye diseases. Whether the content of these two microbes in each disease correlates with the severity of eye disease needs to be further studied.

### Interactions Between Microbes

**Figure 4:**
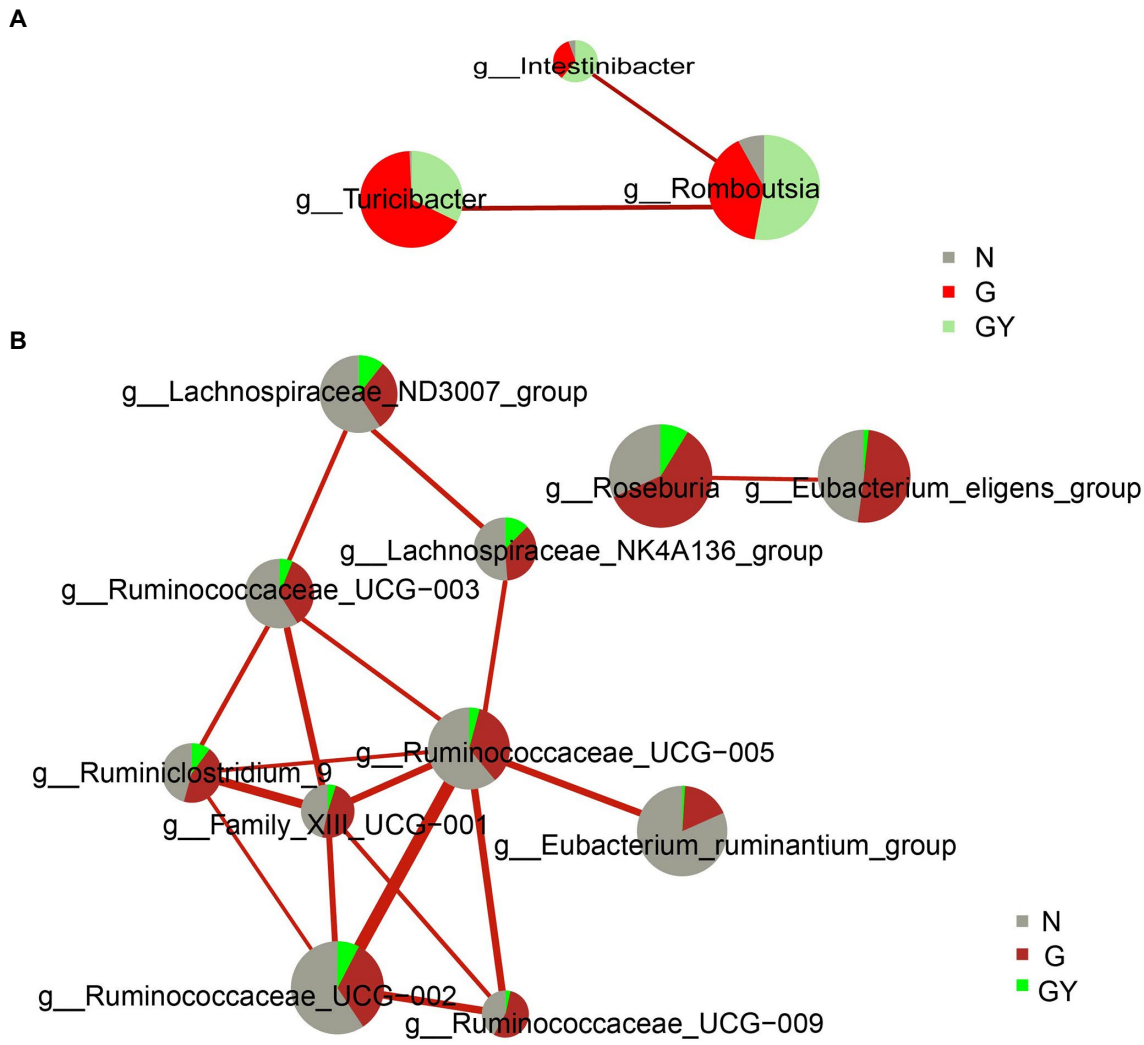
Interaction network diagram of microbes related to Group G **(A)** and Group GY **(B)**. Red represents a positive correlation. The thicker the line is, the stronger the correlation is. The group with a larger sector area has a higher content of this microbe.

To better understand the role of these microbes, we analyzed the interaction of these significantly changed microbes using the personalbio platform and identified three modules. The first is the module associated with active anterior scleritis, which included *Intestinibacter*, *Romboutsia*, and *Turicibacter*; all were enriched in both the G and GY groups and correlated positively with each other. This suggests that these microbes participate in the pathogenesis of noninfectious scleritis by promoting each other (Figure 4A).

The second module is associated with RA or the shared pathogenesis pathway of scleritis and RA, including the following nine microbes with decreased contents: *Family_XIII_UCG-001*, *Eubacterium_ruminantium_group*, *Ruminococcus_UCG-002*, *Ruminococcus_UCG-003*, *Ruminococcus_UCG-005*, *Ruminococcus_UCG-009*, *Lachnospiraceae_NK4A136_group*, *Lachnospiraceae_ND3007_group*, and *Ruminiclostridium_9*. These microbes were decreased in both the GY and G groups; however, the difference was significant only in the former. Hence, these microbes may play a protective role in the pathogenesis of RA or the shared pathogenesis pathway of scleritis and RA. In addition, *Ruminococcace_UCG-005* and *Family_XIII_UCG-001* were at the center of the association network and closely associated with other microbes. Therefore, we consider that these two microbes may play a key role in the whole mechanism of action (Figure 4B).

The third module included two microbes (*Eubacterium_eligens_group*, *Roseburia*), which only exhibited significantly decreased contents in the GY group and were negatively related to each other. The final consequence of the mutual negative regulation between the two microbes appears to be related to the pathogenesis of RA or the shared pathogenesis pathway of scleritis and RA (Figure 4B).

## Discussion

At the genus level, compared with healthy controls, noninfectious anterior scleritis patients without rheumatoid arthritis showed 14 enriched and 10 decreased microbes, whereas noninfectious anterior scleritis patients with rheumatoid arthritis showed 13 enriched microbes and 18 decreased microbes. Among them, four bacterial genera exhibited the same changes between the G and GY groups. This result suggests that these four genera may be related to noninfectious anterior scleritis. *Lachnospiraceae_ND3007_group* and *Eggerthella* are detected in both anterior scleritis and active-onset VKH patients, indicating that they may be nonspecifically involved in various immune-related eye diseases. The biomarkers of each group were analyzed by LEFse, and the interaction between these microbes was explored by a correlation diagram.

By analyzing α- and β-diversity, we found a significant decrease in species richness in the GY group compared with the G and N groups, indicating more severe intestinal dysbiosis in the GY group. This finding suggests that such changes in intestinal microbes may be closely related to the pathogenesis of active anterior scleritis with RA. However, whether the change in intestinal microbes is associated with the severity of scleritis needs to be further evaluated.

**Figure 5:**
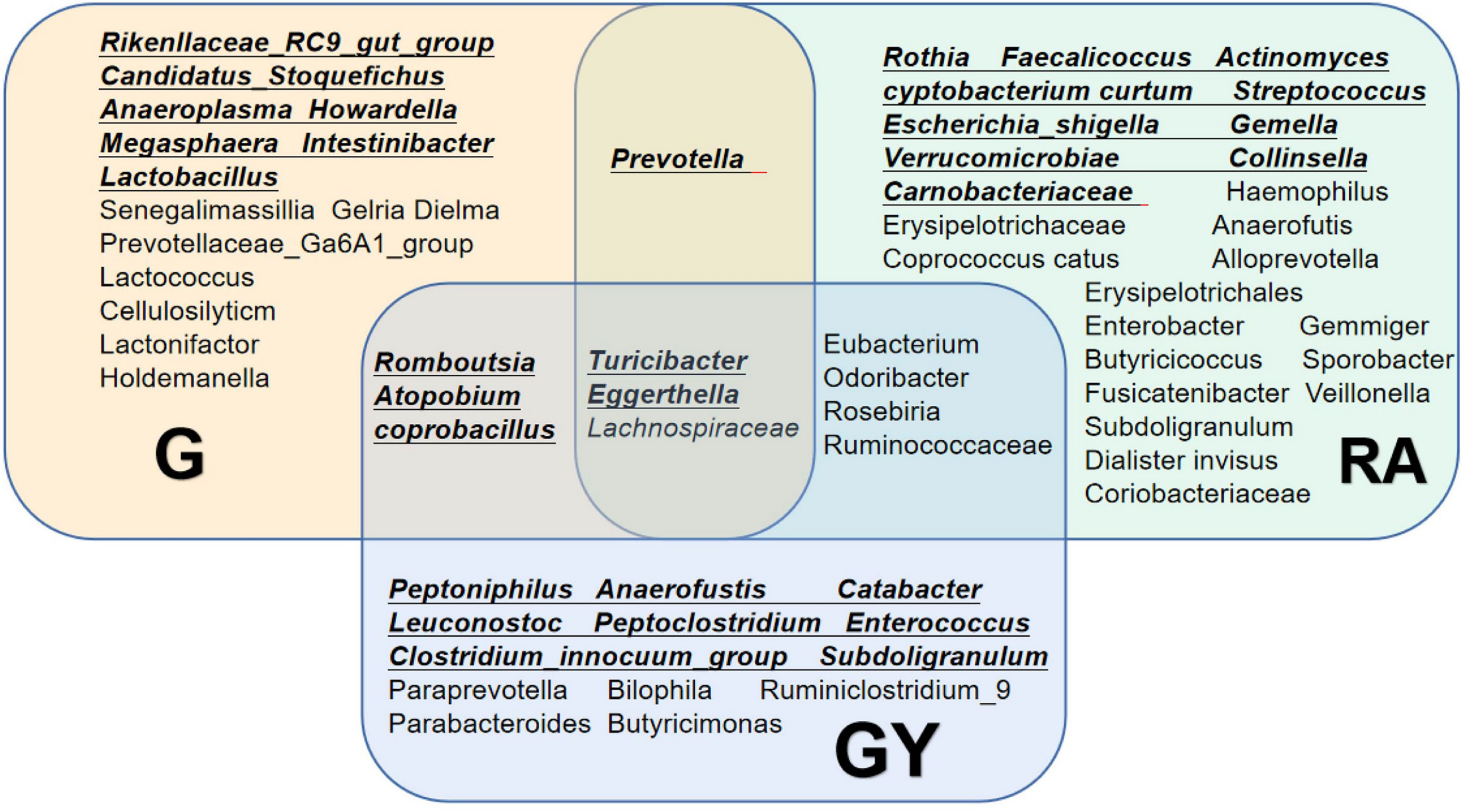
Changes in the gut microbiome in the G and GY groups and patients with rheumatoid arthritis. Underlined intestinal microbes exhibited increased abundances, and microbes without underlining exhibited decreased abundances.

To explore microbes related to scleritis, we referred to a series of studies on changes in intestinal microbes in patients with RA (Figure 5; de Oliveira et al., 2017; Maeda and Takeda, 2017, 2019; Forbes et al., 2018; Xu et al., 2020; Chu et al., 2021; Reyes-Castillo et al., 2021). Comparing these reported microbes with our results, *Turicibacter* and *Eggerthella* enrichment and *Lachnospiraceae* reduction were found in Groups G and GY and patients with RA. Therefore, these microbes may be involved in the pathogenesis of both scleritis and RA. Increases in *Romboutsia*, *Atopobium* and *Coprobacillus* were found in both Groups G and GY but not in patients with RA, indicating that these microbes may be closely related to the pathogenesis of scleritis. As decreased abundances of *Eubacterium*, *Odoribacter*, *Roseburia*, and *Ruminococcaceae* were observed in GY group and RA patients, these microbes may be closely related to the pathogenesis of RA.

The mechanisms of these microbes involved in the occurrence of scleritis are not completely clear. It has been proven that increases in IL-1β, TNF-α and IL-6 play a key role in the pathogenesis of scleritis and that biological agents such as antitumor necrosis factor agents and interleukin-1 and interleukin-6 inhibitors control scleral inflammation either in an idiopathic manner or in a background of immune-mediated systemic disorders (Sota et al., 2021). *Romboutsia* and *Turicibacter* are positively related to IL-6, IL-1β, TNF-α, IL-23 and IFN-γ (Bosshard et al., 2002; Munyaka et al., 2016; Li et al., 2020). These cytokines can lead to an increase in macrophages and B cells, which are important in the pathogenesis of scleritis (Nishio et al., 2021). In addition, TNF-α elevates the level of MMPs, which may disrupt the balance between MMPs (MMP3 and MMP9) and TIMPs and induce scleritis (Wakefield et al., 2013; Vergouwen et al., 2020). Our results show *Romboutsia* and *Turicibacter* to be enriched and promote each other in active anterior scleritis patients with and without RA. Hence, these two microbes may participate in the pathogenesis of scleritis by increasing inflammatory cytokines, which in turn induce a subsequent immune response. *Lactococcus lactis* is also related to inflammatory cytokines. Simčič et al. found that *L. lactis* effectively downregulates the TNF-α response (Simcic et al., 2019). In patients with active anterior scleritis, decreased *Lactococcus* indicates reduced ability to downregulate TNF-α, which in turn causes disease *via* relatively elevated TNF-α levels. Enriched *Atopobium* was found in both the G and GY groups. It has been reported that *Atopobium* is an H2S-producing bacterium (Yang and Jobin, 2017), and an increase in H2S can exacerbate intestinal epithelial barrier damage (Ye et al., 2018). In our patients, enriched *Atopobium* may be involved in the pathogenesis of scleritis through this mechanism.

The mechanism by which these microbes became decreased only in Group GY, and not in Group G (Type C), is related to SCFAs. Previous studies have shown that a decrease in SCFA levels is closely related to the pathogenesis of RA (Yang and Cong, 2021). *Odoribacter* (Turna et al., 2020), *Roseburia* (Tamanai-Shacoori et al., 2017), and *Ruminiclostridium_9* (Hsiao et al., 2021) are butyrate producers (butyrate is a type of SCFA); *Ruminococcaceae* (Kang et al., 2017) and *Eubacterium* (Li et al., 2022) are short-chain fatty acid (SCFA) producers. In our patients, the abundances of all these microbes were decreased, as was SCFA production. However, whether the decrease in SCFA also promotes the occurrence of scleritis needs to be further explored.

An increase in *Eggerthella* and a decrease in *Lachnospiraceae* were found in both the G and GY groups, with the changes in the latter being more severe. *Lachnospiraceae* is an SCFA producer, and the decreased abundance of *Lachnospiraceae_ND3007_group* may have decreased SCFA production (Kang et al., 2017). *Eggerthella* enrichment might induce inflammatory cytokines, including TNF-α, IL-1β, and IL-6 (Li et al., 2022). An increase in *Eggerthella* and a decrease in *Lachnospiraceae* have also been found in other immune-mediated diseases, including Vogt–Koyanagi–Harada (VKH; Li et al., 2022) Crohn’s disease (CD), ulcerative colitis (UC) and multiple sclerosis (MS; Forbes et al., 2018). This result suggests that the roles of *Eggerthella* and *Lachnospiraceae* are not specific. The content of *Lachnospiraceae_ND3007_group* was lower and that of *Eggerthella* higher in the GY group. Whether the levels of these two microbes are related to the severity of eye disease needs to be further studied.

Our study suggests the existence of a gut-eye axis. Intestinal dysbiosis may be a crucial factor influencing ocular diseases. Dysbiosis of the intestinal microbiota causes upregulated expression of inflammatory cytokines in peripheral blood, which in turn causes ocular inflammation. In addition, the gut microbiota may cause extraintestinal diseases, including RA, through antigenic mimicry (Pianta et al., 2017). These are potential pathways or mechanisms by which dysbiosis induces autoimmunity and the link with scleritis or RA-associated physiopathology.

In conclusion, our study indicates that intestinal microbes are involved in the pathogenesis of noninfectious anterior scleritis in patients with and without rheumatoid arthritis. The roles of these microbes are both pathogenic and protective. They may participate in the pathogenesis of anterior scleritis by interacting with each other and altering immunity.

## Limitation

This is a descriptive study about changes in intestinal microbes in noninfectious anterior scleritis patients with and without RA, and there is a lack of research on the mechanisms by which these altered intestinal microbes cause scleritis. As there is currently no accepted animal model of anterior scleritis, it is not feasible to validate the mechanisms of these intestinal microbes. *In vitro* studies of the effects of these intestinal microbes on patient’s immune cells might provide some clues. Additionally, whether the extent of these changed microbes correlates with the severity of scleritis requires further investigation.

## Data Availability Statement

The original contributions presented in the study are included in the article/supplementary material, further inquiries can be directed to the corresponding author.

## Ethics Statement

This study was approved by the Ethics Committee of the Second Hospital of Jilin University. The number is 2021120. The patients/participants provided their written informed consent to participate in this study.

## Author Contributions

ML: data collection and analysis and writing the manuscript. LY, FB, and LZ: collection of samples, data analysis, and editing the manuscript. XL: acquisition of funding, supervision and planning of experiments, data analysis, and writing the manuscript. All authors contributed to the article and approved the submitted version.

## Funding

This work was supported by the National Natural Science Foundation of China, grant no. 81300752, the Jilin Province Science and Technology Development Plan Project, grant no. 20200201333JC, and the Jilin Province Health Special Project, grant no. 2020SCZT058.

## Conflict of Interest

The authors declare that the research was conducted in the absence of any commercial or financial relationships that could be construed as a potential conflict of interest.

## Publisher’s Note

All claims expressed in this article are solely those of the authors and do not necessarily represent those of their affiliated organizations, or those of the publisher, the editors and the reviewers. Any product that may be evaluated in this article, or claim that may be made by its manufacturer, is not guaranteed or endorsed by the publisher.

## Abbreviations

ANCA: Antineutrophil cytoplasmic antibody
HLA-DR: Human leukocyte antigen DR
RA: Rheumatoid arthritis
DNA: Deoxyribonucleic acid
RNA: Ribonucleic acid
16S rRNA: 16S Ribosomal RNA
OTU: Operational taxonomic unit
ACE: Abundance-based coverage estimator
LDA: Linear discriminant analysis
LEFse: LDA effect Size
AMOVA: Analysis of Molecular Variance
VKH: Vogt–Koyanagi–Harada Syndrome
MMPs: Matrix metalloproteinases
TIMPs: Tissue inhibitor of matrix metalloproteinases
H2S: Hydrogen sulfide
SCFA: Short-chain fatty acid
CD: Crohn’s disease
UC: Ulcerative colitis
MS: Multiple sclerosis
G: Patients with active noninfectious anterior scleritis
GY: Patients with active noninfectious anterior scleritis combined with active rheumatoid arthritis
N: Healthy controls without immune-mediated diseases

## References

[ref1] Aletaha D. Neogi T. Silman A. J. Funovits J. Felson D. T. Bingham C. O. 3rd. (2010). 2010 rheumatoid arthritis classification criteria: an American College of Rheumatology/European League Against Rheumatism collaborative initiative. Ann. Rheum. Dis. 69, 1580–1588. doi: 10.1136/ard.2010.138461, PMID: 20699241

[ref2] Bosshard P. P. Zbinden R. Altwegg M. (2002). Turicibacter sanguinis gen. nov., sp. nov., a novel anaerobic, Gram-positive bacterium. Int. J. Syst. Evol. Microbiol. 52, 1263–1266. doi: 10.1099/00207713-52-4-1263, PMID: 12148638

[ref3] Chu X. J. Cao N. W. Zhou H. Y. Meng X. Guo B. Zhang H. Y.. (2021). The oral and gut microbiome in rheumatoid arthritis patients: a systematic review. Rheumatology 60, 1054–1066. doi: 10.1093/rheumatology/keaa835, PMID: 33450018

[ref4] de Oliveira G. L. V. Leite A. Z. Higuchi B. S. Gonzaga M. I. Mariano V. S. (2017). Intestinal dysbiosis and probiotic applications in autoimmune diseases. Immunology 152, 1–12. doi: 10.1111/imm.12765, PMID: 28556916 PMC5543467

[ref5] Dutta Majumder P. Agrawal R. McCluskey P. Biswas J. (2020). Current approach for the diagnosis and management of noninfective scleritis. Asia Pac. J. Ophthalmol. 10, 212–223. doi: 10.1097/APO.0000000000000341 33290287

[ref6] Forbes J. D. Chen C. Y. Knox N. C. Marrie R. A. El-Gabalawy H. de Kievit T.. (2018). A comparative study of the gut microbiota in immune-mediated inflammatory diseases-does a common dysbiosis exist? Microbiome 6:221. doi: 10.1186/s40168-018-0603-4, PMID: 30545401 PMC6292067

[ref7] Hsiao Y. P. Chen H. L. Tsai J. N. Lin M. Y. Liao J. W. Wei M. S.. (2021). Administration of *Lactobacillus reuteri* combined with *Clostridium butyricum* attenuates Cisplatin-induced renal damage by gut microbiota reconstitution, increasing butyric acid production, and suppressing renal inflammation. Nutrients 13:28. doi: 10.3390/nu13082792, PMID: 34444952 PMC8402234

[ref8] Kang C. Wang B. Kaliannan K. Wang X. Lang H. Hui S.. (2017). Gut microbiota mediates the protective effects of dietary capsaicin against chronic low-grade inflammation and associated obesity induced by high-fat diet. MBio 8:9. doi: 10.1128/mBio.00900-17 PMC5442453 28536285

[ref9] Karami J. Aslani S. Jamshidi A. Garshasbi M. Mahmoudi M. (2019). Genetic implications in the pathogenesis of rheumatoid arthritis; an updated review. Gene 702, 8–16. doi: 10.1016/j.gene.2019.03.033 30904715

[ref10] Li M. Yang L. Cao J. Liu T. Liu X. (2022). Enriched and decreased intestinal microbes in active VKH patients. Invest. Ophthalmol. Vis. Sci. 63:21. doi: 10.1167/iovs.63.2.21, PMID: 35142786 PMC8842635

[ref11] Li R. Yao Y. Gao P. Bu S. (2020). The therapeutic efficacy of Curcumin vs. metformin in modulating the gut microbiota in NAFLD rats: A comparative study. Front. Microbiol. 11:555293. doi: 10.3389/fmicb.2020.555293 33584555 PMC7874275

[ref12] Maeda Y. Takeda K. (2017). Role of gut microbiota in rheumatoid arthritis. J. Clin. Med. 6:3. doi: 10.3390/jcm6060060, PMID: 28598360 PMC5483870

[ref13] Maeda Y. Takeda K. (2019). Host-microbiota interactions in rheumatoid arthritis. Exp. Mol. Med. 51, 1–6. doi: 10.1038/s12276-019-0283-6, PMID: 31827063 PMC6906371

[ref14] Munyaka P. M. Rabbi M. F. Khafipour E. Ghia J. E. (2016). Acute dextran sulfate sodium (DSS)-induced colitis promotes gut microbial dysbiosis in mice. J. Basic Microbiol. 56, 986–998. doi: 10.1002/jobm.201500726, PMID: 27112251

[ref15] Nishio Y. Taniguchi H. Takeda A. Hori J. (2021). Immunopathological analysis of a mouse model of arthritis-associated scleritis and implications for molecular targeted therapy for severe scleritis. Int. J. Mol. Sci. 23, 12–13. doi: 10.3390/ijms23010341, PMID: 35008766 PMC8745222

[ref16] Pianta A. Arvikar S. L. Strle K. Drouin E. E. Wang Q. Costello C. E.. (2017). Two rheumatoid arthritis-specific autoantigens correlate microbial immunity with autoimmune responses in joints. J. Clin. Invest. 127, 2946–2956. doi: 10.1172/JCI93450, PMID: 28650341 PMC5531397

[ref17] Promelle V. Goeb V. Gueudry J. (2021). Rheumatoid arthritis associated Episcleritis and Scleritis: An update on treatment perspectives. J. Clin. Med. 10:1. doi: 10.3390/jcm10102118, PMID: 34068884 PMC8156434

[ref18] Reyes-Castillo Z. Valdes-Miramontes E. Llamas-Covarrubias M. Munoz-Valle J. F. (2021). Troublesome friends within us: the role of gut microbiota on rheumatoid arthritis etiopathogenesis and its clinical and therapeutic relevance. Clin. Exp. Med. 21, 1–13. doi: 10.1007/s10238-020-00647-y, PMID: 32712721

[ref19] Simcic S. Berlec A. Stopinsek S. Strukelj B. Orel R. (2019). Engineered and wild-type *L. lactis* promote anti-inflammatory cytokine signalling in inflammatory bowel disease patient's mucosa. World J. Microbiol. Biotechnol. 35:45. doi: 10.1007/s11274-019-2615-z, PMID: 30810891

[ref20] Sota J. Girolamo M. M. Frediani B. Tosi G. M. Cantarini L. Fabiani C. (2021). Biologic therapies and small molecules for the management of non-infectious scleritis: a narrative review. Ophthalmol. Ther. 10, 777–813. doi: 10.1007/s40123-021-00393-8, PMID: 34476773 PMC8589879

[ref21] Tamanai-Shacoori Z. Smida I. Bousarghin L. Loreal O. Meuric V. Fong S. B.. (2017). Roseburia spp.: a marker of health? Future Microbiol. 12, 157–170. doi: 10.2217/fmb-2016-0130 28139139

[ref22] Turna J. Grosman Kaplan K. Anglin R. Patterson B. Soreni N. Bercik P.. (2020). The gut microbiome and inflammation in obsessive-compulsive disorder patients compared to age- and sex-matched controls: a pilot study. Acta Psychiatr. Scand. 142, 337–347. doi: 10.1111/acps.13175, PMID: 32307692

[ref23] Vergouwen D. P. C. Rothova A. Berge J. C. T. Verdijk R. M. van Laar J. A. M. Vingerling J. R.. (2020). Current insights in the pathogenesis of scleritis. Exp. Eye Res. 197:108078. doi: 10.1016/j.exer.2020.108078, PMID: 32504648

[ref24] Vignesh A. P. Srinivasan R. (2015). Ocular manifestations of rheumatoid arthritis and their correlation with anti-cyclic citrullinated peptide antibodies. Clin. Ophthalmol. 9, 393–397. doi: 10.2147/OPTH.S77210, PMID: 25750517 PMC4348132

[ref25] Wakefield D. Di Girolamo N. Thurau S. Wildner G. McCluskey P. (2013). Scleritis: Immunopathogenesis and molecular basis for therapy. Prog. Retin. Eye Res. 35, 44–62. doi: 10.1016/j.preteyeres.2013.02.004, PMID: 23454614

[ref26] Xu H. Zhao H. Fan D. Liu M. Cao J. Xia Y.. (2020). Interactions between gut microbiota and immunomodulatory cells in rheumatoid arthritis. Mediators Inflamm. 2020, 1–14. doi: 10.1155/2020/1430605 PMC7499318 32963490

[ref27] Yang W. Cong Y. (2021). Gut microbiota-derived metabolites in the regulation of host immune responses and immune-related inflammatory diseases. Cell. Mol. Immunol. 18, 866–877. doi: 10.1038/s41423-021-00661-4, PMID: 33707689 PMC8115644

[ref28] Yang Y. Jobin C. (2017). Novel insights into microbiome in colitis and colorectal cancer. Curr. Opin. Gastroenterol. 33, 422–427. doi: 10.1097/MOG.0000000000000399, PMID: 28877044 PMC5826583

[ref29] Ye Z. Zhang N. Wu C. Zhang X. Wang Q. Huang X.. (2018). A metagenomic study of the gut microbiome in Behcet's disease. Microbiome 6:135. doi: 10.1186/s40168-018-0520-6, PMID: 30077182 PMC6091101

